# Supplementary material for: Tissue vs. Fecal-Derived Bacterial Dysbiosis in Precancerous Colorectal Lesions: A Systematic Review
Source: Cancers (Basel). 2023 Mar 4;15(5):1602. doi: 10.3390/cancers15051602 (PMC10000868; doi:10.3390/cancers15051602)
Supplement: Supplementary file 1 [file cancers-15-01602-s001.zip › Table S2.pdf]

Supplementary Table 2. Quality assessment of tissue + stool or stool or tissue-based case-control, cross sectional and cohort studies using Newcastle-Ottawa scale. A study can be awarded a maximum of one star for each numbered item within the Selection and Exposure categories. A maximum of two stars can be given for Comparability.

	Case Definition (Histologically confirmed)	Representativeness of cases (n=100+)	Selection of controls (from same area)	Definition of controls (Histological confirmation)	Comparability (Matched controls or same tissue area)	Exposure measurement (used internal control)	Same method for cases and controls	Non-response rate (Described or used consecutive recruitment)	Total score
**Tissue + Stool**									
Zeller et al. (2014) [30]	1	1	0	0	1	1	1	0	5
Mira-Pascual et al. (2015) [23]	1	0	1	1	2	1	1	0	7
Yu et al. (2015) [25]	1	1	1	0	0	1	1	0	5
Flemer et al. (2017) [24]	1	0	1	1	2	0	1	0	6
Shen et al. (2021) [26]	1	1	1	1	0	1	1	1	7
Watson et al. (2021) [27]	1	0	1	1	0	1	1	0	5
Avelar-Barragan et al. (2022) [31]	1	0	1	0	0	1	1	1	5
**Stool**									
Brim et al. (2013) [18]	1	0	1	1	0	1	1	0	5
Chen et al. (2013) [49]	1	0	1	1	2	0	0	0	5
Feng et al. (2015) [50]	1	0	1	1	2	1	1	0	7
Goedert et al. (2015) [37]	1	0	1	0	0	1	1	1	5
Kasai et al. (2016) [55]	1	0	1	0	0	1	1	1	5
Peters et al. (2016) [51]	1	1	1	1	0	1	1	1	7
Hale et al. (2017) [35]	1	1	1	0	0	1	1	0	5
Yang et al. (2019) [38]	1	1	1	1	0	1	1	0	6
Clos-Garcia et al. (2020) [32]	1	1	1	1	0	1	1	1	7
Wei et al. (2020) [33]	1	0	1	0	0	1	1	1	5
Zhang, He et al. (2022) [39]	1	0	1	0	0	1	1	1	5
Hua et al. (2022) [40]	1	1	1	0	0	1	1	0	5
Bosch et al. (2022) [34]	1	0	1	0	2	1	1	0	6
Zhang, Lu et al. (2022) [52]	1	1	1	0	0	1	1	1	6
**Tissue**									
Sanapareddy et al. (2012) [41]	1	0	1	1	0	1	1	0	5
Dejea et al. (2014) [42]	1	0	0	1	1	1	1	0	5
Geng et al. (2014) [43]	1	0	1	1	1	1	1	0	6
Nugent et al. (2014) [36]	1	0	1	1	1	1	1	0	6
Lu et al. (2016) [44]	1	0	1	1	2	1	1	0	7
Yu et al. (2016) [54]	1	1	1	0	1	1	1	0	6
Xu et al. (2017) [45]	1	1	1	1	0	1	1	0	6
Wachsmannova et al. (2018) [46]	1	0	1	1	0	1	1	0	5
Bundgaard-Nielsen et al.(2019) [47]	1	1	1	1	0	1	1	1	7
Wang et al. (2020) [53]	1	0	1	1	0	1	1	0	5
Liu et al. (2021) [48]	1	0	1	1	0	1	1	0	5
