# Supplementary material for: Tissue vs. Fecal-Derived Bacterial Dysbiosis in Precancerous Colorectal Lesions: A Systematic Review
Source: Cancers (Basel). 2023 Mar 4;15(5):1602. doi: 10.3390/cancers15051602 (PMC10000868; doi:10.3390/cancers15051602)
Supplement: Supplementary file 1 [file cancers-15-01602-s001.zip › Table S3.pdf]

Supplementary Table 3. Summary of human studies investigating precancerous colorectal lesions and healthy control stool and tissue specimens addressing microbial compositional shifts.

**Author (publish date)**	**Quality assessment (NOS) ≥5/9**	**Study group size (n)**	**Control group size (n)**	**Type of matrix (F/T)**	**Detection method**	**Prevalence/ abundance of bacteria (Phylum; class; order; family; genus, species) and/or α-/β-diversity in precancerous CR lesions**	**Clinical evidence (association with A, Cis and/or CRC)**
Human studies examining **FECAL** and/vs. **TISSUE** – derived gut bacterial composition in precancerous colorectal lesions (and/or CRC)							
Zeller et al. (2014) [30]	5/9	French (Fr) cohort: TA: 42; CRC: 53; German cohort (G): CRC: 38; German cohort (G): CRC 48 (at the time of surgery)	Fr: HC: 61; A <1 cm: 27; German, Danish and Spanish cohort (H): HC: 297 (not confirmed by colonoscopy)	Fr and G: F (prior to bowel prep or 10 days after colonoscopy); H: F; G: T (from tumor and matched normal T)	16S, metagenomic sequencing	*Bacteroidetes*: *Firmicutes* ratio and *Ruminococcus* genus differed significantly in A vs. CRC and HC.	Microbiota changes during early stages of neoplastic growth suggesting that identification of reliable microbial markers for AA as CRC precursors may be possible.
Mira-Pascual et al. (2015) [23]	7/9	TA: 11; CRC: 7	HC: 10 (F and normal rectal T)	F (prior to bowel prep); T (from tumor)	16S: V1–V3 PCoA; *Fn* qPCR	F: ↑ *Blautia, Fusobacterium*. T: ↑*Bifidobacterium*, *Fusobacterium* (*Fn* and others), *Enterobacteriaceae, Akkermansia, Blautia, Prevotella, Bacteroides* in A < CRC.	Microbial changes according to disease progression step and tumor severity. T samples represented the underlying dysbiosis, whereas F

NOS: Newcastle-Ottawa scale; NA: not applicable; A: adenoma; AA: advanced adenoma (> 1 cm in diameter and/or high grade dysplasia (+/- villous, or tubulovillous morphology)); NAA: non-advanced adenoma; TA or CA: conventional or traditional adenoma (≤ 1 cm diameter, without dysplasia (tubular, tubulovillous or villous)); HP: hyperplastic polyp; SSA: sessile serrated adenoma; TSA: traditional serrated adenoma; Cis: Ca in situ/intramucosal carcinoma/carcinoma in adenoma; CR: colorectal; CRA: colorectal adenoma; CRC: colorectal cancer; LST: laterally spreading tumor; HC: healthy controls; F: fecal samples; T: tissue (mucosal) samples; 16S: 16s rRNA gene sequencing; WGS: whole-genome shotgun sequencing; T-RFLP: terminal restriction fragment length polymorphism; NGS: next-generation sequencing; MGWAS: metagenome-wide association study; FISH: fluorescence in situ hybridization; LC–TOFMS: liquid chromatography time-of-flight mass spectrometry; GC–TOFMS: gas chromatography time-of-flight mass spectrometry; HPLC: high-performance liquid chromatography analysis; UPLC–MS: ultra performance liquid chromatography-mass spectrometry; ITS: Internal Transcribed Spacer (ITS) ribosomal RNA sequencing; FOBT: fecal occult blood test; CI: confidence interval; OR: odds ratio; AUC: area under the curve; OUTs: operational taxonomic units; *ETBF*: enterotoxigenic *Bacteroides fragilis*; SCFAs: short-chain fatty acids; FFFFT: fresh-frozen formalin fixed tissue; FFPE: formalin-fixed paraffin-embedded; prep: preparation; *Fn: Fusobacterium nucleatum*.

						↑*Enterococcaceae* family in A > CRC > HC.	samples seem not to be appropriate to detect shifts in microbial composition. *Fusobacterium, Bacteroides, and Methanobacteriales* may provide a potential marker for early detection of CRC.
Yu et al. (2015) [25]	5/9	F: A: 47; CRC: 42; T: A: 30; CRC: 31	F: HC: 52; T: HC: 37	F (prior to bowel prep); T (left colonic biopsies –during CR surgery)	16S; 454 FLX pyrosequencing, *Fn* qPCR	F: ↑ *Fusobacterium, Escherichia-Shigella, Coprococcus, Streptococcus, Enterococcus spp.;* ↓ *Actinomyces, Bifidobacterium, Lactobacillus,* butyrate-producing bacteria (*Clostridium, Roseburia, Eubacterium, Blautia, and Dorea spp*.) during the A-carcinoma sequence. F and T: ↑ *Fusobacterial* phylum: from HC < A < CRC	Microbial structures were altered in the lumen and the mucosa during the progression of the A-carcinoma sequence. *Fusobacterium* expression in the T was consistent with that in F; therefore, F samples may replace tissue specimens as a simpler and more practical diagnostic method for the early detection of *Fusobacterium* enrichment.
Flemer et al. (2017) [24]	6/9	A: 21 (T samples); CRC: 59 (32 both F and T samples)	HC: 56 (32 age-matched)	F (prior to bowel prep) and T (CRC group: 'ON' and 'OFF' the tumor, proximal and distal after surgery;	16S; qRT-PCR	F and T: microbiota differed in CRC vs. HC (p<0.05) and A vs. HC (p > 0.05). T: A had similar trends to CRC changes (p > 0.05).	Alterations not restricted to the cancerous tissue. Differences between distal and proximal CRC. The microbiota compositional differences in patients with CRC are not secondary to the cancer per se.

NOS: Newcastle-Ottawa scale; NA: not applicable; A: adenoma; AA: advanced adenoma (> 1 cm in diameter and/or high grade dysplasia (+/- villous, or tubulovillous morphology)); NAA: non-advanced adenoma; TA or CA: conventional or traditional adenoma (≤ 1 cm diameter, without dysplasia (tubular, tubulovillous or villous)); HP: hyperplastic polyp; SSA: sessile serrated adenoma; TSA: traditional serrated adenoma; Cis: Ca in situ/intramucosal carcinoma/carcinoma in adenoma; CR: colorectal; CRA: colorectal adenoma; CRC: colorectal cancer; LST: laterally spreading tumor; HC: healthy controls; F: fecal samples; T: tissue (mucosal) samples; 16S: 16s rRNA gene sequencing; WGS: whole-genome shotgun sequencing; T-RFLP: terminal restriction fragment length polymorphism; NGS: next-generation sequencing; MGWAS: metagenome-wide association study; FISH: fluorescence in situ hybridization; LC–TOFMS: liquid chromatography time-of-flight mass spectrometry; GC–TOFMS: gas chromatography time-of-flight mass spectrometry; HPLC: high-performance liquid chromatography analysis; UPLC–MS: ultra performance liquid chromatography-mass spectrometry; ITS: Internal Transcribed Spacer (ITS) ribosomal RNA sequencing; FOBT: fecal occult blood test; CI: confidence interval; OR: odds ratio; AUC: area under the curve; OUTs: operational taxonomic units; *ETBF*: enterotoxigenic *Bacteroides fragilis*; SCFAs: short-chain fatty acids; FFFFT: fresh-frozen formalin fixed tissue; FFPE: formalin-fixed paraffin-embedded; prep: preparation; *Fn: Fusobacterium nucleatum*.

				A: undiseased proximal and distal colon mucosa)<br>No F samples from A group		No difference was found in microbiota composition of tumor and paired non-tumor tissues.	F microbiota only partially reflected mucosal microbiota.<br>T microbiota in A similar to CRC (not statistically significant)
Shen et al. (2021) [26]	7/9	T group: A: 8; LST: 11.<br>F group: A: 208; LST: 109; CRC: 45	T: HC: 5;<br>F: HC: 113	F (prior to bowel prep); T (from tumor)	16S; qPCR	T: ↑ genus *Lactobacillus-Streptococcus* and the *spp*. *ETBF–Peptostreptococcus stomatis (P. stomatis)–Parvimonas micra (P. micra*); *Lactobacillus johnsonil (L. johnsonil)* in LST.<br>*F: ETBF, P. stomatis*, and *P. micra* steadily ↑ in LST and CRC.	F microbial biomarkers *ETBF–P. stomatis–P. micra* were defined as early noninvasive biomarkers of LST.<br>*P. stomatis* behaved high accuracy on predicting A recurrence after LST resections.
Watson et al. (2021) [27]	5/9	A: 48	Non-A patients: 56	F; T (from polyp, normal right, left colon and rectum); oral swab (O)	16S: V4	T and F: ↑ *Firmicutes*, *Bacteroidetes*; O: ↑ *Firmicutes, Proteobacteria*.<br>T: ↑ microbial diversity > F and O<br>T:↑ families *Lachnospiraceae, Ruminococcaceae* genera *Bacteroides* and *Marvinbryantia, Blautia obeum, Streptococcus,* genera *Veillonella, Odoribacter, Haemophillus, Coprobacter, Eggerthella, Granulicatella, Actinomyces* in A.	F- and T-associated microbiomes are distinct; T microbiome is highly predictive of A status.

NOS: Newcastle-Ottawa scale; NA: not applicable; A: adenoma; AA: advanced adenoma (> 1 cm in diameter and/or high grade dysplasia (+/- villous, or tubulovillous morphology)); NAA: non-advanced adenoma; TA or CA: conventional or traditional adenoma (≤ 1 cm diameter, without dysplasia (tubular, tubulovillous or villous)); HP: hyperplastic polyp; SSA: sessile serrated adenoma; TSA: traditional serrated adenoma; Cis: Ca in situ/intramucosal carcinoma/carcinoma in adenoma; CR: colorectal; CRA: colorectal adenoma; CRC: colorectal cancer; LST: laterally spreading tumor; HC: healthy controls; F: fecal samples; T: tissue (mucosal) samples; 16S: 16s rRNA gene sequencing; WGS: whole-genome shotgun sequencing; T-RFLP: terminal restriction fragment length polymorphism; NGS: next-generation sequencing; MGWAS: metagenome-wide association study; FISH: fluorescence in situ hybridization; LC–TOFMS: liquid chromatography time-of-flight mass spectrometry; GC–TOFMS: gas chromatography time-of-flight mass spectrometry; HPLC: high-performance liquid chromatography analysis; UPLC–MS: ultra performance liquid chromatography-mass spectrometry; ITS: Internal Transcribed Spacer (ITS) ribosomal RNA sequencing; FOBT: fecal occult blood test; CI: confidence interval; OR: odds ratio; AUC: area under the curve; OUTs: operational taxonomic units; *ETBF*: enterotoxigenic *Bacteroides fragilis*; SCFAs: short-chain fatty acids; FFFFT: fresh-frozen formalin fixed tissue; FFPE: formalin-fixed paraffin-embedded; prep: preparation; *Fn: Fusobacterium nucleatum*.

						F:↑ family *Lachnospiraceae*, taxa *Erysipelatoclostridium* ramosum in A.<br>O: ↑ *Rothia mucilaginosa* in A.	
Avelar-Barragan et al. (2022) [31]	5/9	TA: 45;<br>SP (HP, TSA, or SSP): 33	HC: 50	F (stool samples 4-6 weeks after colonoscopy); T (mucosal brush samples from polyp and healthy opposite wall; mucosal aspirates from near the polyp; lavage aspirates)	16S, ITS sequencing; WGS	↑ *Gemellaceae* family and 2 *Streptococcus spp*. in mucosal aspirates vs. mucosal brushes.<br>↑ diversity in F samples vs. mucosal aspirates, marginally ↑ diversity > in lavage aspirates.<br>F: 63% *Firmicutes*, 27% *Bacteroides*, 3.5% *Actinobacteria*, 4.5% *Proteobacteria*.<br>Mucosal aspirates and lavage aspirates: 73 and 75% *Firmicutes*, 15 and 11% *Bacteroides*, 4.7 and 5.2% *Actinobacteria*, and 4.0 and 6.6% *Proteobacteria*, respectively.<br>TA mucosal aspirates: ↑*Lachnospiraceae,* such as *Ruminococcus gnavus, C. scindens, Bacteroides fragilis;*	Microbiomes of mucosal brushes and mucosal aspirates did not significantly differ in diversity or composition. Microbiomes of F samples were significantly ↑ diverse and compositionally distinct vs. mucosal aspirates.<br>Mucosal samples are sensitive enough to study the microbiome of CRA found within the proximal colon.

NOS: Newcastle-Ottawa scale; NA: not applicable; A: adenoma; AA: advanced adenoma (> 1 cm in diameter and/or high grade dysplasia (+/- villous, or tubulovillous morphology)); NAA: non-advanced adenoma; TA or CA: conventional or traditional adenoma (≤ 1 cm diameter, without dysplasia (tubular, tubulovillous or villous)); HP: hyperplastic polyp; SSA: sessile serrated adenoma; TSA: traditional serrated adenoma; Cis: Ca in situ/intramucosal carcinoma/carcinoma in adenoma; CR: colorectal; CRA: colorectal adenoma; CRC: colorectal cancer; LST: laterally spreading tumor; HC: healthy controls; F: fecal samples; T: tissue (mucosal) samples; 16S: 16s rRNA gene sequencing; WGS: whole-genome shotgun sequencing; T-RFLP: terminal restriction fragment length polymorphism; NGS: next-generation sequencing; MGWAS: metagenome-wide association study; FISH: fluorescence in situ hybridization; LC–TOFMS: liquid chromatography time-of-flight mass spectrometry; GC–TOFMS: gas chromatography time-of-flight mass spectrometry; HPLC: high-performance liquid chromatography analysis; UPLC–MS: ultra performance liquid chromatography-mass spectrometry; ITS: Internal Transcribed Spacer (ITS) ribosomal RNA sequencing; FOBT: fecal occult blood test; CI: confidence interval; OR: odds ratio; AUC: area under the curve; OUTs: operational taxonomic units; *ETBF*: enterotoxigenic *Bacteroides fragilis*; SCFAs: short-chain fatty acids; FFFFT: fresh-frozen formalin fixed tissue; FFPE: formalin-fixed paraffin-embedded; prep: preparation; *Fn: Fusobacterium nucleatum*.

						SP mucosal aspirates: ↓ *E. lenta, A. hadrus*<br>HC vs TA mucosal aspirates: *Ruthenibactrium sp., Ruminococcus gnavus, Ruminococcus sp., Dorea sp., and Blautia sp.*<br>HC vs SP: *Anaerostipes hadrus, Dorea longiatena, E. lenta, Clostridium ramosum, and Alistipes finegoldii;*<br>SP vs TA: *Gemmiger formicilis, E. lenta, Bifidobacterium sp., Ruthenibacterium sp., UBA7182 HGM12585.*	
Human studies examining **FECAL** – derived gut bacterial composition in precancerous colorectal lesions (and/or CRC)							
Brim et al. (2013) [18]	5/9	A: 6	HC: 6	F (2 months after colonoscopy)	16S, Human intestinal Tract Chip, 454 pyrosequencing	Subgenus level: ↑ *Bacteroides* in HC vs A;<br>↑ *Firmicutes* in A vs HC.<br>*Bacteroidetes* and *Firmicutes* – most ↑groups in all samples.	Bacteria and associated functions within the *Bacteroides* group need to be further analyzed for potential actors in the early colon oncogenic transformation in a large sample size.

NOS: Newcastle-Ottawa scale; NA: not applicable; A: adenoma; AA: advanced adenoma (> 1 cm in diameter and/or high grade dysplasia (+/- villous, or tubulovillous morphology)); NAA: non-advanced adenoma; TA or CA: conventional or traditional adenoma (≤ 1 cm diameter, without dysplasia (tubular, tubulovillous or villous)); HP: hyperplastic polyp; SSA: sessile serrated adenoma; TSA: traditional serrated adenoma; Cis: Ca in situ/intramucosal carcinoma/carcinoma in adenoma; CR: colorectal; CRA: colorectal adenoma; CRC: colorectal cancer; LST: laterally spreading tumor; HC: healthy controls; F: fecal samples; T: tissue (mucosal) samples; 16S: 16s rRNA gene sequencing; WGS: whole-genome shotgun sequencing; T-RFLP: terminal restriction fragment length polymorphism; NGS: next-generation sequencing; MGWAS: metagenome-wide association study; FISH: fluorescence in situ hybridization; LC–TOFMS: liquid chromatography time-of-flight mass spectrometry; GC–TOFMS: gas chromatography time-of-flight mass spectrometry; HPLC: high-performance liquid chromatography analysis; UPLC–MS: ultra performance liquid chromatography-mass spectrometry; ITS: Internal Transcribed Spacer (ITS) ribosomal RNA sequencing; FOBT: fecal occult blood test; CI: confidence interval; OR: odds ratio; AUC: area under the curve; OUTs: operational taxonomic units; *ETBF*: enterotoxigenic *Bacteroides fragilis*; SCFAs: short-chain fatty acids; FFFFT: fresh-frozen formalin fixed tissue; FFPE: formalin-fixed paraffin-embedded; prep: preparation; *Fn: Fusobacterium nucleatum*.

Chen et al. (2013) [49]	5/9	AA: 47 (sex- and age matched)	HC: 47	F	16S	↓ Butyrate/butyrate-producing bacteria; ↓ *Clostridium*, *Roseburia*, *Eubacteria*; ↑ *Enterococcus*, *Bacteroidetes*, *Streptococcus spp*. in AA.	A high-fiber dietary pattern, subsequent consistent production of SCFAs and healthy gut microbiota are associated with ↓ risk of AA.
Feng et al. (2015) [50]	7/9	AA: 44, CRC: 46 (45-86 yr, both genders and white race)	HC: 57	F (prior to bowel prep)	MGWAS	*Bacteroides, Prevotella, Parabacteroides spp. Alistipes putredinis, Bilophila wadsworthia, Lachnospiraceae bacterium, Fusobacterium, E. coli*. ↑ *B. dorei*, B. *massiliensis* from HC→AA, and significant ↑↑↑ of *B. massiliensis, B. ovatus, B. vulgatus* and *E. coli* from AA→to CRC. *B. dorei*, *B. vulgatus*, *E. coli* also correlated with levels of CRP. No difference in the abundance of *Akkermansia* among AA, HC and CRC.	Development of AA and CRC.
Goedert et al. (2015) [37]	5/9	A: 20; CRC: 2; other: 15	HC: 24	F (during FIT+ screening colonoscopy)	16S	Phylum-level F community composition differed significantly between A and HC (P = 0.02).	If confirmed in larger, more diverse populations, F microbiota analysis might be employed to improve screening for CRA.

NOS: Newcastle-Ottawa scale; NA: not applicable; A: adenoma; AA: advanced adenoma (> 1 cm in diameter and/or high grade dysplasia (+/- villous, or tubulovillous morphology)); NAA: non-advanced adenoma; TA or CA: conventional or traditional adenoma (≤ 1 cm diameter, without dysplasia (tubular, tubulovillous or villous)); HP: hyperplastic polyp; SSA: sessile serrated adenoma; TSA: traditional serrated adenoma; Cis: Ca in situ/intramucosal carcinoma/carcinoma in adenoma; CR: colorectal; CRA: colorectal adenoma; CRC: colorectal cancer; LST: laterally spreading tumor; HC: healthy controls; F: fecal samples; T: tissue (mucosal) samples; 16S: 16s rRNA gene sequencing; WGS: whole-genome shotgun sequencing; T-RFLP: terminal restriction fragment length polymorphism; NGS: next-generation sequencing; MGWAS: metagenome-wide association study; FISH: fluorescence in situ hybridization; LC–TOFMS: liquid chromatography time-of-flight mass spectrometry; GC–TOFMS: gas chromatography time-of-flight mass spectrometry; HPLC: high-performance liquid chromatography analysis; UPLC–MS: ultra performance liquid chromatography-mass spectrometry; ITS: Internal Transcribed Spacer (ITS) ribosomal RNA sequencing; FOBT: fecal occult blood test; CI: confidence interval; OR: odds ratio; AUC: area under the curve; OUTs: operational taxonomic units; *ETBF*: enterotoxigenic *Bacteroides fragilis*; SCFAs: short-chain fatty acids; FFFFT: fresh-frozen formalin fixed tissue; FFPE: formalin-fixed paraffin-embedded; prep: preparation; *Fn: Fusobacterium nucleatum*.

						Rank phylum-level abundance distinguished A from HC. A prevalence was 59% in phylum-level cluster B versus 20% in cluster A. ↑↑↑ Proteobacteria (*Pseudomonas, Escherichia, Shigella, Salmonella, Serratia, Klebsiella, and Helicobacter*), ↑ TM7; ↓ *Fusobacteria*.	
Kasai et al. (2016) [55]	5/9	A: 50, CRC: 9 (3 - invasive and 6 - Cis)	HC: 49	F (prior to bowel prep)	T-RFLP; NGS	T-RFLP: no significant differences in bacterial population between HC, A and CRC. NGS: ↑ *F. varium* rDNA copies in Cis vs HC; Genera: *Actinomyces, Atopobium, Fusobacterium, and Haemophilus, Actinomyces odontolyticus, Bacteroides fragile, Clostridium nexile, Fusobacterium varium, Haemophilus parainfluenzae, Prevotella stercorea, Streptococcus*	Gut microbiota is related to CRC prevention and development.

NOS: Newcastle-Ottawa scale; NA: not applicable; A: adenoma; AA: advanced adenoma (> 1 cm in diameter and/or high grade dysplasia (+/- villous, or tubulovillous morphology)); NAA: non-advanced adenoma; TA or CA: conventional or traditional adenoma (≤ 1 cm diameter, without dysplasia (tubular, tubulovillous or villous)); HP: hyperplastic polyp; SSA: sessile serrated adenoma; TSA: traditional serrated adenoma; Cis: Ca in situ/intramucosal carcinoma/carcinoma in adenoma; CR: colorectal; CRA: colorectal adenoma; CRC: colorectal cancer; LST: laterally spreading tumor; HC: healthy controls; F: fecal samples; T: tissue (mucosal) samples; 16S: 16s rRNA gene sequencing; WGS: whole-genome shotgun sequencing; T-RFLP: terminal restriction fragment length polymorphism; NGS: next-generation sequencing; MGWAS: metagenome-wide association study; FISH: fluorescence in situ hybridization; LC–TOFMS: liquid chromatography time-of-flight mass spectrometry; GC–TOFMS: gas chromatography time-of-flight mass spectrometry; HPLC: high-performance liquid chromatography analysis; UPLC–MS: ultra performance liquid chromatography-mass spectrometry; ITS: Internal Transcribed Spacer (ITS) ribosomal RNA sequencing; FOBT: fecal occult blood test; CI: confidence interval; OR: odds ratio; AUC: area under the curve; OUTs: operational taxonomic units; *ETBF*: enterotoxigenic *Bacteroides fragilis*; SCFAs: short-chain fatty acids; FFFFT: fresh-frozen formalin fixed tissue; FFPE: formalin-fixed paraffin-embedded; prep: preparation; *Fn: Fusobacterium nucleatum*.

						gordonii, *Veillonella dispar* significantly associated with Cis.	
Peters et al. (2016) [51]	7/9	CA: 144 (proximal: 87; distal: 55; NAA: 121; AA: 22), SA: 73 (HP: 40; SSA: 33)	HC: 323	F (prior to bowel prep or min. 5 days after colonoscopy)	16S	↓*Clostridia* (families *Ruminococcaceae, Clostridiaceae*, and *Lachnospiraceae*); ↑classes of *Bacilli*, *Gammaproteobacteria*, (order *Enterobacteriales*), genera *Actinomyces, Streptococcus* in CA. ↓ richness in CA vs. HC; ↓↓ in AA ↓ *Erysipelotrichi* class in SSA vs. HP and HC.	Gut microbes may play a role in the early stages of CR carcinogenesis through the development of CAs.
Hale et al. (2017) [35]	5/9	A (> 1cm): 233	HC: 547	F (prior to bowel prep)	16S	↑ *Bacteroidetes* phyla, *Deltaproteobacteria* class, OTUs in the *Bilophila, Desulfovibrio, Sutterella,* and *Mogibacterium* genera.	*Bilophilia* and *Desulfovibrio* may produce genotoxic or inflammatory metabolites such as H2S and secondary bile acids, which could play a role in catalyzing A development and eventually CRC.
Yang et al. (2019) [38]	6/9	A: 117, CRC: 62	HC: 104	F (prior to bowel prep)	16S: V3-4	↑enterotypes: *Bacteroides*, *Prevotella*, *Escherichia*. ↓*Oscillospira* in AA → ↓↓ *Oscillospira* in stage 0 CRC;	F microbiota differs along the A-carcinoma sequence and across enterotypes. ↓ CAG cluster 5 and cluster 7, composed primarily of butyrate-

NOS: Newcastle-Ottawa scale; NA: not applicable; A: adenoma; AA: advanced adenoma (> 1 cm in diameter and/or high grade dysplasia (+/- villous, or tubulovillous morphology)); NAA: non-advanced adenoma; TA or CA: conventional or traditional adenoma (≤ 1 cm diameter, without dysplasia (tubular, tubulovillous or villous)); HP: hyperplastic polyp; SSA: sessile serrated adenoma; TSA: traditional serrated adenoma; Cis: Ca in situ/intramucosal carcinoma/carcinoma in adenoma; CR: colorectal; CRA: colorectal adenoma; CRC: colorectal cancer; LST: laterally spreading tumor; HC: healthy controls; F: fecal samples; T: tissue (mucosal) samples; 16S: 16s rRNA gene sequencing; WGS: whole-genome shotgun sequencing; T-RFLP: terminal restriction fragment length polymorphism; NGS: next-generation sequencing; MGWAS: metagenome-wide association study; FISH: fluorescence in situ hybridization; LC–TOFMS: liquid chromatography time-of-flight mass spectrometry; GC–TOFMS: gas chromatography time-of-flight mass spectrometry; HPLC: high-performance liquid chromatography analysis; UPLC–MS: ultra performance liquid chromatography-mass spectrometry; ITS: Internal Transcribed Spacer (ITS) ribosomal RNA sequencing; FOBT: fecal occult blood test; CI: confidence interval; OR: odds ratio; AUC: area under the curve; OUTs: operational taxonomic units; *ETBF*: enterotoxigenic *Bacteroides fragilis*; SCFAs: short-chain fatty acids; FFFFT: fresh-frozen formalin fixed tissue; FFPE: formalin-fixed paraffin-embedded; prep: preparation; *Fn: Fusobacterium nucleatum*.

						↓ *Haemophilus* in stage 0 CRC → ↓↓ *Haemophilus* in early-stage CRC. ↑ *Fusobacterium*, *Enterococcus*, *Aeromonas*, ↓ *Eubacterium, Roseburia, Faecalibacterium, Oscillospira* from A → CRC.	producing bacteria, is a suitable marker of CRC.
Clos-Garcia et al. (2020) [32]	7/9	AA: 69; CRC: 99	HC: 77	F	16S: V1–V2, targeted UPLC-MS metabolomics	↑ *Adlercreutzia* in AA. ↓ *Firmicutes* phylum and ↓*Firmicutes: Bacteroidetes* ratio in AA and CRC. ↓↓↓ *Fusobacteria* phylum in AA and HC. No significant differences in the genera abundance of F microbiome between AA and HC.	Integration of metabolomics and microbiome data revealed tight interactions between bacteria and host and performed better than FOB test for CRC diagnosis.
Wei et al. (2020) [33]	5/9	A: 43; iFOBT+: 36	HC: 53	F	16S: V3-4, short- and long-read sequencing	↑ *Klebsiella pneumonia, Fusobacterium varium, Fusobacterium mortiferum* in A vs. iFOBT+ and HC.	Identification of adenomatous polyp-associated microbiomes could potentially function as an auxiliary biomarker for predicting CRC development.
Zhang, He et al.	5/9	A: 29; CRC: 30	HC: 35	F (before colonoscopy)	shotgun metagenomi	*Clostridium Bolteae, Hungatella Hathewayi, Eggerthella lenta*	*Peptostreptococcus stomatis, Clostridium symbiosum, Hungatella*

NOS: Newcastle-Ottawa scale; NA: not applicable; A: adenoma; AA: advanced adenoma (> 1 cm in diameter and/or high grade dysplasia (+/- villous, or tubulovillous morphology)); NAA: non-advanced adenoma; TA or CA: conventional or traditional adenoma (≤ 1 cm diameter, without dysplasia (tubular, tubulovillous or villous)); HP: hyperplastic polyp; SSA: sessile serrated adenoma; TSA: traditional serrated adenoma; Cis: Ca in situ/intramucosal carcinoma/carcinoma in adenoma; CR: colorectal; CRA: colorectal adenoma; CRC: colorectal cancer; LST: laterally spreading tumor; HC: healthy controls; F: fecal samples; T: tissue (mucosal) samples; 16S: 16s rRNA gene sequencing; WGS: whole-genome shotgun sequencing; T-RFLP: terminal restriction fragment length polymorphism; NGS: next-generation sequencing; MGWAS: metagenome-wide association study; FISH: fluorescence in situ hybridization; LC–TOFMS: liquid chromatography time-of-flight mass spectrometry; GC–TOFMS: gas chromatography time-of-flight mass spectrometry; HPLC: high-performance liquid chromatography analysis; UPLC–MS: ultra performance liquid chromatography-mass spectrometry; ITS: Internal Transcribed Spacer (ITS) ribosomal RNA sequencing; FOBT: fecal occult blood test; CI: confidence interval; OR: odds ratio; AUC: area under the curve; OUTs: operational taxonomic units; *ETBF*: enterotoxigenic *Bacteroides fragilis*; SCFAs: short-chain fatty acids; FFFFT: fresh-frozen formalin fixed tissue; FFPE: formalin-fixed paraffin-embedded; prep: preparation; *Fn: Fusobacterium nucleatum*.

(2022) [39]					c sequencing	presented consistent changes in A and CRC vs. HC. ↑ *Blautia hansenii, Streptococcus sanguinis, Enterococcus faecalis, and Oxalobacter formigenes* in A. Correlations of *Parvimonas micra* with *Peptostreptococcus stomatis, Eggerthella lenta* with *Lactobacillus mucosae, Hungatela hathawayi* with *Ruthenibacterium lactatiformans* in A and CRC.	*hathewayi, Parvimonas micra, and Gemella Morbillorum* identified as a diagnostic model to identify CRC patients.
Hua et al. (2022) [40]	5/9	A: 20; CRC: 154	HC: 199	F (prior to colonoscopy)	16S	Genus level: ↑*Acidaminococcus, Alloprevotella, Mycoplasma, Sphingobacterium*; ↓ *Acidaminococcus* with the order of HC → A→ CRC ($P < 0.05$). ↑ *Parvimonas, Peptostreptococcus, Prevotella, Butyricimonas, Alistipes, Odoribacter* in A and CRC. *Butyricimonas synergistica, Agrobacterium larrymoorei, Bacteroides plebeius, Lachnospiraceae bacterium feline oral taxon 001,*	Several intestinal bacteria changed along the A-carcinoma sequence and might be the potential markers for the diagnosis and treatment of CRA/CRC.

NOS: Newcastle-Ottawa scale; NA: not applicable; A: adenoma; AA: advanced adenoma (> 1 cm in diameter and/or high grade dysplasia (+/- villous, or tubulovillous morphology); NAA: non-advanced adenoma; TA or CA: conventional or traditional adenoma (≤ 1 cm diameter, without dysplasia (tubular, tubulovillous or villous)); HP: hyperplastic polyp; SSA: sessile serrated adenoma; TSA: traditional serrated adenoma; Cis: Ca in situ/intramucosal carcinoma/carcinoma in adenoma; CR: colorectal; CRA: colorectal adenoma; CRC: colorectal cancer; LST: laterally spreading tumor; HC: healthy controls; F: fecal samples; T: tissue (mucosal) samples; 16S: 16s rRNA gene sequencing; WGS: whole-genome shotgun sequencing; T-RFLP: terminal restriction fragment length polymorphism; NGS: next-generation sequencing; MGWAS: metagenome-wide association study; FISH: fluorescence in situ hybridization; LC–TOFMS: liquid chromatography time-of-flight mass spectrometry; GC–TOFMS: gas chromatography time-of-flight mass spectrometry; HPLC: high-performance liquid chromatography analysis; UPLC–MS: ultra performance liquid chromatography-mass spectrometry; ITS: Internal Transcribed Spacer (ITS) ribosomal RNA sequencing; FOBT: fecal occult blood test; CI: confidence interval; OR: odds ratio; AUC: area under the curve; OUTs: operational taxonomic units; *ETBF*: enterotoxigenic *Bacteroides fragilis*; SCFAs: short-chain fatty acids; FFFFT: fresh-frozen formalin fixed tissue; FFPE: formalin-fixed paraffin-embedded; prep: preparation; *Fn: Fusobacterium nucleatum*.

						Clostridium scindens, Prevotella heparinolytica, bacterium LD2013, Streptococcus mutans, Lachnospiraceae bacterium 19gly4, Eubacterium hallii - best performance in distinguishing A from CRC (AUC = 85.54%, 95% CI: 78.83-92.25%).	
Bosch et al. (2022) [34]	6/9	A: 32 (19 strictly matched on age, BMI and smoking habits: AA: 9; NAA: 10)	HC: 32	F (1 week prior to bowel prep for colonoscopy and 3 months after polypectomy)	16S: V4; HPLC	↑ *Butyricimonasspp., Catenibacterium spp., Faecalitalea spp.*; ↓ *Anaerostipes spp., Bifidobacterium spp., Cyanobacteria* within the *Gastranaerophilales* order in A vs. HC.	F microbiome of post-endoscopy patients resemble those, of controls. A-specific panels of amino acids may improve the effectiveness of the surveillance program by detection of high-risk individuals for earlier surveillance endoscopy timing, leading to less unnecessary endoscopies and less interval cancer.
Zhang, Lu et al. (2022) [52]	6/9	AA: 268; NAA: 490	HC: 788	F	16S	No significant differences in the α-diversity among the 3 groups. ↑ Genera: *Fusobacterium, Tyzzerella 4, Phascolarctobacterium, Clostridium sensu stricto 1; Streptococcus,*	Identified microbial signatures could complement FITs for detecting AA. Gut microbiota can act as a promising tool to optimize the current CRC screening modalities.

NOS: Newcastle-Ottawa scale; NA: not applicable; A: adenoma; AA: advanced adenoma (> 1 cm in diameter and/or high grade dysplasia (+/- villous, or tubulovillous morphology)); NAA: non-advanced adenoma; TA or CA: conventional or traditional adenoma (≤ 1 cm diameter, without dysplasia (tubular, tubulovillous or villous)); HP: hyperplastic polyp; SSA: sessile serrated adenoma; TSA: traditional serrated adenoma; Cis: Ca in situ/intramucosal carcinoma/carcinoma in adenoma; CR: colorectal; CRA: colorectal adenoma; CRC: colorectal cancer; LST: laterally spreading tumor; HC: healthy controls; F: fecal samples; T: tissue (mucosal) samples; 16S: 16s rRNA gene sequencing; WGS: whole-genome shotgun sequencing; T-RFLP: terminal restriction fragment length polymorphism; NGS: next-generation sequencing; MGWAS: metagenome-wide association study; FISH: fluorescence in situ hybridization; LC–TOFMS: liquid chromatography time-of-flight mass spectrometry; GC–TOFMS: gas chromatography time-of-flight mass spectrometry; HPLC: high-performance liquid chromatography analysis; UPLC–MS: ultra performance liquid chromatography-mass spectrometry; ITS: Internal Transcribed Spacer (ITS) ribosomal RNA sequencing; FOBT: fecal occult blood test; CI: confidence interval; OR: odds ratio; AUC: area under the curve; OUTs: operational taxonomic units; *ETBF*: enterotoxigenic *Bacteroides fragilis*; SCFAs: short-chain fatty acids; FFFFT: fresh-frozen formalin fixed tissue; FFPE: formalin-fixed paraffin-embedded; prep: preparation; *Fn: Fusobacterium nucleatum*.

						Gemella, Actinomyces, Terrisporobacter in AA vs. HC	
Human studies examining **TISSUE** – derived gut bacterial composition in precancerous colorectal lesions (and/or CRC)							
Sanapareddy et al. (2012) [41]	5/9	A: 33	A-free controls: 38	T (from normal rectal mucosa – 10-12 cm from anal verge)	16S, 454 pyrosequencing	↑ numbers of bacteria from 87 taxa in A comparing to A-free controls. ↑ *TM7, Cyanobacteria, Verrucomicrobia, Acidovorax, Aquabacterium, Cloacibacterium, Helicobacter, Lactococcus, Lactobacillus, Pseudomonas* and other (phylum *Proteobacteria*)	Sequence analysis of the microbiota could be used to identify patients at risk for developing A.
Dejea et al. (2014) [42]	5/9	USA and Malaysian cohorts: Right-sided: A: 6; CRC: 15; Left-sided: A: 2; CRC:15.	HC: 22 (11 right and left-matched pairs, none biofilm positive, USA cohort); paired normal adjacent tissue (at the time of surgery/colonoscopy)	T (FFFT from tumor)	16S: V3–V5, high-throughput sequencing, FISH	Patients with biofilm-positive tumors (A or CRC), all had biofilms on their tumor-free mucosa far distant from their tumors. ↑ *Bacteroidetes* and *Firmicutes* (family *Lachnospiraceae* including *Clostridium*, *Ruminococcus*, and *Butyrivibrio*) in A and CRC. ↑ *Fusobacteria*, *Gammaproteobacteria* (*Enterobacteriaceae* family) in CRC.	Biofilm presence correlates with bacterial tissue invasion and changes in tissue biology with ↑ cellular proliferation. Colon mucosal biofilm detection may predict ↑ risk for development of sporadic CRC.

NOS: Newcastle-Ottawa scale; NA: not applicable; A: adenoma; AA: advanced adenoma (> 1 cm in diameter and/or high grade dysplasia (+/- villous, or tubulovillous morphology)); NAA: non-advanced adenoma; TA or CA: conventional or traditional adenoma (≤ 1 cm diameter, without dysplasia (tubular, tubulovillous or villous)); HP: hyperplastic polyp; SSA: sessile serrated adenoma; TSA: traditional serrated adenoma; Cis: Ca in situ/intramucosal carcinoma/carcinoma in adenoma; CR: colorectal; CRA: colorectal adenoma; CRC: colorectal cancer; LST: laterally spreading tumor; HC: healthy controls; F: fecal samples; T: tissue (mucosal) samples; 16S: 16s rRNA gene sequencing; WGS: whole-genome shotgun sequencing; T-RFLP: terminal restriction fragment length polymorphism; NGS: next-generation sequencing; MGWAS: metagenome-wide association study; FISH: fluorescence in situ hybridization; LC–TOFMS: liquid chromatography time-of-flight mass spectrometry; GC–TOFMS: gas chromatography time-of-flight mass spectrometry; HPLC: high-performance liquid chromatography analysis; UPLC–MS: ultra performance liquid chromatography-mass spectrometry; ITS: Internal Transcribed Spacer (ITS) ribosomal RNA sequencing; FOBT: fecal occult blood test; CI: confidence interval; OR: odds ratio; AUC: area under the curve; OUTs: operational taxonomic units; *ETBF*: enterotoxigenic *Bacteroides fragilis*; SCFAs: short-chain fatty acids; FFFFT: fresh-frozen formalin fixed tissue; FFPE: formalin-fixed paraffin-embedded; prep: preparation; *Fn: Fusobacterium nucleatum*.

						Biofilms identified on surgically resected, normal tissues were also consistently diverse, composed of *Bacteroidetes, Lachnospiraceae, Gammaproteobacteria.*	
Geng et al. (2014) [43]	6/9	A:10; CRC: 8	HC: 10 (location-matched)	T (from tumor)	16S, 454 pyrosequencing	↑ *Enterobacteriaceae, Enterobacter, Pseudomonadaceae, Neisseriaceae, Chryseobacterium, NKB19, Planomicrobium* (potential driver bacteria)*;* ↓ *Anoxybacillus, Megamonas, Streptophyta, Microbacterium, Methylobacterium, TM7-3, Staphylococcus*, etc. – (potential anti-inflammatory passenger bacteria) in A vs. HC and CRC.	Bacterial driver-passenger model for CRC.
Nugent et al. (2014) [36]	6/9	A: 15	A-free controls: 15	T (from normal rectal mucosa – 10–12 cm from the anal verge)	qPCR; LC−TOFMS, GC−TOFMS	↑ *Bifidobacterium* and *Eubacteria;* ↑ *Escherichia coli, Clostridium sp., Bacteroides sp* (without statistical significance) in A vs. A-free controls.	Metabolic products of bacteria and the interplay between bacteria and metabolites is important in the development of CRA and CRC.

NOS: Newcastle-Ottawa scale; NA: not applicable; A: adenoma; AA: advanced adenoma (> 1 cm in diameter and/or high grade dysplasia (+/- villous, or tubulovillous morphology)); NAA: non-advanced adenoma; TA or CA: conventional or traditional adenoma (≤ 1 cm diameter, without dysplasia (tubular, tubulovillous or villous)); HP: hyperplastic polyp; SSA: sessile serrated adenoma; TSA: traditional serrated adenoma; Cis: Ca in situ/intramucosal carcinoma/carcinoma in adenoma; CR: colorectal; CRA: colorectal adenoma; CRC: colorectal cancer; LST: laterally spreading tumor; HC: healthy controls; F: fecal samples; T: tissue (mucosal) samples; 16S: 16s rRNA gene sequencing; WGS: whole-genome shotgun sequencing; T-RFLP: terminal restriction fragment length polymorphism; NGS: next-generation sequencing; MGWAS: metagenome-wide association study; FISH: fluorescence in situ hybridization; LC−TOFMS: liquid chromatography time-of-flight mass spectrometry; GC−TOFMS: gas chromatography time-of-flight mass spectrometry; HPLC: high-performance liquid chromatography analysis; UPLC–MS: ultra performance liquid chromatography-mass spectrometry; ITS: Internal Transcribed Spacer (ITS) ribosomal RNA sequencing; FOBT: fecal occult blood test; CI: confidence interval; OR: odds ratio; AUC: area under the curve; OUTs: operational taxonomic units; *ETBF*: enterotoxigenic *Bacteroides fragilis*; SCFAs: short-chain fatty acids; FFFFT: fresh-frozen formalin fixed tissue; FFPE: formalin-fixed paraffin-embedded; prep: preparation; *Fn: Fusobacterium nucleatum*.

Lu et al. (2016) [44]	7/9	A: 31	HC: 20; paired normal adjacent tissue	T (from adenoma)	16S pyrosequencing	Abundance of 8 phyla (*Firmicutes, Proteobacteria, Bacteroidetes, Actinobacteria, Chloroflexi, Cyanobacteria, Candidate-division TM7,* and *Tenericutes*) was significantly different in A and HC. ↑ *Lactococcus*, *Pseudomonas*; ↓ *Enterococcus*, *Bacillus*, *Solibacillus* in A. Genera: ↑ *Proteobacteria* in A.	Suggesting CR preneoplastic lesion may be the most important factor leading to alterations in bacterial community composition.
Yu et al. (2016) [54]	6/9	Proximal HP: 35, SSA: 33; Distal HP: 40; Proximal TA: 38; Distal TA: 41; Distal CRC: 45 (+ 10 metastatic, 10 nonmetastatic matched lymph nodes); Proximal CRC: 48 (+ 10 metastatic, 10	HC: 20 (10 proximal, 10 distal CR mucosa)	T (from tumor)	16S, FISH, *Fn* PCR	↑ *Fusobacterium* in proximal HPs and SSAs vs. proximal TAs and distal TAs ($p < 0.05$).	Invasive *Fn* is involved primarily in the carcinogenesis of proximal colon cancers that develop along the serrated neoplasia pathway, having only a minor role in the traditional A-carcinoma sequence.

NOS: Newcastle-Ottawa scale; NA: not applicable; A: adenoma; AA: advanced adenoma (> 1 cm in diameter and/or high grade dysplasia (+/- villous, or tubulovillous morphology)); NAA: non-advanced adenoma; TA or CA: conventional or traditional adenoma (≤ 1 cm diameter, without dysplasia (tubular, tubulovillous or villous)); HP: hyperplastic polyp; SSA: sessile serrated adenoma; TSA: traditional serrated adenoma; Cis: Ca in situ/intramucosal carcinoma/carcinoma in adenoma; CR: colorectal; CRA: colorectal adenoma; CRC: colorectal cancer; LST: laterally spreading tumor; HC: healthy controls; F: fecal samples; T: tissue (mucosal) samples; 16S: 16s rRNA gene sequencing; WGS: whole-genome shotgun sequencing; T-RFLP: terminal restriction fragment length polymorphism; NGS: next-generation sequencing; MGWAS: metagenome-wide association study; FISH: fluorescence in situ hybridization; LC–TOFMS: liquid chromatography time-of-flight mass spectrometry; GC–TOFMS: gas chromatography time-of-flight mass spectrometry; HPLC: high-performance liquid chromatography analysis; UPLC–MS: ultra performance liquid chromatography-mass spectrometry; ITS: Internal Transcribed Spacer (ITS) ribosomal RNA sequencing; FOBT: fecal occult blood test; CI: confidence interval; OR: odds ratio; AUC: area under the curve; OUTs: operational taxonomic units; *ETBF*: enterotoxigenic *Bacteroides fragilis*; SCFAs: short-chain fatty acids; FFFFT: fresh-frozen formalin fixed tissue; FFPE: formalin-fixed paraffin-embedded; prep: preparation; *Fn: Fusobacterium nucleatum*.

		nonmetastatic matched lymph nodes).					
Xu et al. (2017) [45]	6/9	A: 47; CRC: 52	HC: 61	T (from tumor)	16S	↑ *Acidomonas*, *Escherichia*, *Pseudomonas*, *Sphingomonas* in A and HC vs. CRC. Chao1 Richness Index of the mucosal microbiota was significantly different in HC, A, and CRC. Shannon Index and Simpson Index in the 3 groups were not significantly different. No significant difference for any phylum in A vs. HC.	*Butyricicoccus*, *E. coli*, *Fusobacterium* can be used as potential biomarkers for HC, A, and CRC groups, respectively.
Wachsmannova et al. (2018) [46]	5/9	A: 10; CRC: 10	HC: 9; paired nonmalignant tissue	T (4-6 biopsies from tumor)	ENTEROtest 24 plus MALDI-TOF mass spectrometry	↑ intracellular *E. coli* in ↑↑A < ↑↑↑CRC vs. HC. *Escherichia coli, Proteus mirabilis, Proteus vulgaris* in A and CRC. A: ↑ *Pseudomonas aeruginosa, Bacillus cereus, Klebsiella pneumoniae, Enterococcus faecalis.*	Data supports *E. coli's* role as a pro-oncogenic pathogen.

NOS: Newcastle-Ottawa scale; NA: not applicable; A: adenoma; AA: advanced adenoma (> 1 cm in diameter and/or high grade dysplasia (+/- villous, or tubulovillous morphology)); NAA: non-advanced adenoma; TA or CA: conventional or traditional adenoma (≤ 1 cm diameter, without dysplasia (tubular, tubulovillous or villous)); HP: hyperplastic polyp; SSA: sessile serrated adenoma; TSA: traditional serrated adenoma; Cis: Ca in situ/intramucosal carcinoma/carcinoma in adenoma; CR: colorectal; CRA: colorectal adenoma; CRC: colorectal cancer; LST: laterally spreading tumor; HC: healthy controls; F: fecal samples; T: tissue (mucosal) samples; 16S: 16s rRNA gene sequencing; WGS: whole-genome shotgun sequencing; T-RFLP: terminal restriction fragment length polymorphism; NGS: next-generation sequencing; MGWAS: metagenome-wide association study; FISH: fluorescence in situ hybridization; LC–TOFMS: liquid chromatography time-of-flight mass spectrometry; GC–TOFMS: gas chromatography time-of-flight mass spectrometry; HPLC: high-performance liquid chromatography analysis; UPLC–MS: ultra performance liquid chromatography-mass spectrometry; ITS: Internal Transcribed Spacer (ITS) ribosomal RNA sequencing; FOBT: fecal occult blood test; CI: confidence interval; OR: odds ratio; AUC: area under the curve; OUTs: operational taxonomic units; *ETBF*: enterotoxigenic *Bacteroides fragilis*; SCFAs: short-chain fatty acids; FFFFT: fresh-frozen formalin fixed tissue; FFPE: formalin-fixed paraffin-embedded; prep: preparation; *Fn: Fusobacterium nucleatum*.

					Gentamicin-protection assay	*Proteus mirabilis* inside = outside the epithelial cells.	
Bundgaard-Nielsen et al. (2019) [47]	7/9	A: 96; CRC: 99; diverticular disease: 104	Paired normal tissue from CRC group: 76; No HC, no paired normal tissue from A or diverticula groups	T (FFPE from tumor)	16S, *S. gallolyticus, Fn, ETBF* qPCR	*S. gallolyticus* was not detected; ↓ *Fn* and *ETBF* in A vs. CRC and diverticula. *Acinetobacter* genus associated with A and diverticula. Bacterial composition of CRC tissue overlaps with that of paired normal tissue, but differs from A and diverticula.	Findings do not support a role of *Fn* or *ETBF* during CR beginning, while *S. gallolyticus* was not implicated in the CR tissue of a Danish population. A potential role of the bacterial genera *Prevotella* and *Acinetobacter* requires further investigations.
Wang et al. (2020) [53]	5/9	AA: 49	HC: 36; normal adjacent tissue	T (from polyp)	16S: V4, high-throughput sequencing	↑ *Proteobacteria*, ↓ *Firmicutes, Bacteroidetes*; ↑ *Halomonadaceae, Shewanella algae, Lachnospiraceae*; ↓ *Faecalibacterium prausnitzii of Clostridiales, Blautia, Coprococcus of Lachnospiraceae, Bacteroidetes of Bacteroides ovatus* in A vs. HC.	↑ *Halomonadaceae* and *Shewanella algae* and ↓ *Coprococcus* and *Bacteroides ovatus* could serve *as* a biomarker of CRA.

NOS: Newcastle-Ottawa scale; NA: not applicable; A: adenoma; AA: advanced adenoma (> 1 cm in diameter and/or high grade dysplasia (+/- villous, or tubulovillous morphology)); NAA: non-advanced adenoma; TA or CA: conventional or traditional adenoma (≤ 1 cm diameter, without dysplasia (tubular, tubulovillous or villous)); HP: hyperplastic polyp; SSA: sessile serrated adenoma; TSA: traditional serrated adenoma; Cis: Ca in situ/intramucosal carcinoma/carcinoma in adenoma; CR: colorectal; CRA: colorectal adenoma; CRC: colorectal cancer; LST: laterally spreading tumor; HC: healthy controls; F: fecal samples; T: tissue (mucosal) samples; 16S: 16s rRNA gene sequencing; WGS: whole-genome shotgun sequencing; T-RFLP: terminal restriction fragment length polymorphism; NGS: next-generation sequencing; MGWAS: metagenome-wide association study; FISH: fluorescence in situ hybridization; LC–TOFMS: liquid chromatography time-of-flight mass spectrometry; GC–TOFMS: gas chromatography time-of-flight mass spectrometry; HPLC: high-performance liquid chromatography analysis; UPLC–MS: ultra performance liquid chromatography-mass spectrometry; ITS: Internal Transcribed Spacer (ITS) ribosomal RNA sequencing; FOBT: fecal occult blood test; CI: confidence interval; OR: odds ratio; AUC: area under the curve; OUTs: operational taxonomic units; *ETBF*: enterotoxigenic *Bacteroides fragilis*; SCFAs: short-chain fatty acids; FFFFT: fresh-frozen formalin fixed tissue; FFPE: formalin-fixed paraffin-embedded; prep: preparation; *Fn: Fusobacterium nucleatum*.

Liu et al. (2021) [48]	5/9	Cohort 1: Zhongshan Hospital: A: 10, CRC: 11;<br><br>Cohort 2: Fourth Affiliated Hospital: A: 10; CRC: 10;<br>+A: 12; CRC: 15	Paired normal adjacent tissue (2 biopsies)<br>No HC	T (4-6 biopsies from tumor)<br>A	16S: V4	↑ *Fusobacterium*, *Bacteroides*, *Parvimonas*, and *Prevotella* in A → ↑↑ in CRC.<br>A: *Proteobacteria*>> *Firmicutes* -> CRC: *Firmicutes*>> *Proteobacteria*.<br>A and CRC had neoplasia biopsies with significantly different microbiota composition.<br>A and CRC: microbial diversity (α- and β-diversity) between neoplasia and adjacent normal tissue was not significant.	Intra-neoplasia microbiota is heterogeneous and correlates with CR carcinogenesis.<br>Association of intratumoral microbial heterogeneity and CRC-associated genetic alterations of KRAS mutation and MSI.

NOS: Newcastle-Ottawa scale; NA: not applicable; A: adenoma; AA: advanced adenoma (> 1 cm in diameter and/or high grade dysplasia (+/- villous, or tubulovillous morphology)); NAA: non-advanced adenoma; TA or CA: conventional or traditional adenoma (≤ 1 cm diameter, without dysplasia (tubular, tubulovillous or villous)); HP: hyperplastic polyp; SSA: sessile serrated adenoma; TSA: traditional serrated adenoma; Cis: Ca in situ/intramucosal carcinoma/carcinoma in adenoma; CR: colorectal; CRA: colorectal adenoma; CRC: colorectal cancer; LST: laterally spreading tumor; HC: healthy controls; F: fecal samples; T: tissue (mucosal) samples; 16S: 16s rRNA gene sequencing; WGS: whole-genome shotgun sequencing; T-RFLP: terminal restriction fragment length polymorphism; NGS: next-generation sequencing; MGWAS: metagenome-wide association study; FISH: fluorescence in situ hybridization; LC–TOFMS: liquid chromatography time-of-flight mass spectrometry; GC–TOFMS: gas chromatography time-of-flight mass spectrometry; HPLC: high-performance liquid chromatography analysis; UPLC–MS: ultra performance liquid chromatography-mass spectrometry; ITS: Internal Transcribed Spacer (ITS) ribosomal RNA sequencing; FOBT: fecal occult blood test; CI: confidence interval; OR: odds ratio; AUC: area under the curve; OUTs: operational taxonomic units; *ETBF*: enterotoxigenic *Bacteroides fragilis*; SCFAs: short-chain fatty acids; FFFFT: fresh-frozen formalin fixed tissue; FFPE: formalin-fixed paraffin-embedded; prep: preparation; *Fn: Fusobacterium nucleatum*.
